# Prevalence, placenta development, and perinatal outcomes of women with hypertensive disorders of pregnancy at Komfo Anokye Teaching Hospital

**DOI:** 10.1371/journal.pone.0233817

**Published:** 2020-10-29

**Authors:** Stephen Poku Awuah, Isaac Okai, Emmanuel Amankwah Ntim, Kweku Bedu-Addo

**Affiliations:** 1 Department of Physiology, School of Medicine and Dentistry, KNUST, Kumasi, Ghana; 2 Department of Anatomy, School of Medicine and Dentistry, KNUST, Kumasi, Ghana; University of Mississippi Medical Center, UNITED STATES

## Abstract

**Background:**

One of the most common medical problems associated with pregnancy is hypertension. Hypertensive disorders of pregnancy (HDP), which has been attributable to abnormal placentation may have adverse effects on both mother and foetus if left unchecked. The objective of this study was to determine the prevalence of this condition and its effect on placental morphology as well as maternal and perinatal outcomes.

**Materials and methods:**

This was a prospective case-control study, conducted at Komfo Anokye Teaching Hospital (KATH), Ghana between February 2018 and July 2018. The progression of pregnancy in normotensive and hypertensive pregnant women, and the eventual perinatal outcomes were closely followed. Statistical analysis was performed using IMB-SPSS version 23. Associations were considered significant at p values of ≤ 0.05.

**Results:**

From a total of 214 deliveries recorded during the period of study, 84 (39.25%) were hypertensives. Forty four (52%) of the hypertensives had preeclampsia, 28 (33.3%) had gestational hypertension, 6 (7.1%) had eclampsia, 4 (4.8%) had chronic hypertension, and 2 (2.4%) had preeclampsia superimposed on chronic hypertension. The frequency of placental haematoma, placental infarction, and placental calcification in the normotensives were significantly (p = 0.001) lower than that of the hypertensives. The mean placental weight (p = 0.01), placental volume (p = 0.001), placental diameter (p = 0.03), and placental thickness (p = 0.001) of the normotensives were significantly higher than those of the hypertensives. The number of normotensives in whom labour was induced, who had their babies delivered by caesarean section, and who were admitted after they had given birth were significantly (p = 0.001) lower than that of hypertensives who underwent similar procedures. No stillbirths were recorded in the normotensives compared with four in the hypertensives. The number of babies delivered to the normotensives who were admitted to the NICU was significantly (p = 0.001) lower than those delivered by hypertensives.

**Conclusion:**

There was a high prevalence of hypertensive disorders of pregnancy in the study site. Pregnant women who developed HDP are at a risk of developing placental abnormalities that adversely affected perinatal outcomes. These adverse effects can be curtailed by embarking on a vigorous health education drive.

## Introduction

The nurturing and survival of the foetus is dependent on normal physiological changes that are associated with pregnancy. These adaptive changes which are reflected in biochemical parameters are different in the non-pregnant state [1] and become very significant during complications of pregnancy. Hypertension is one of the medical problems that mostly affect pregnant women and it remains an important cause of both maternal and foetal morbidity/mortality. Studies show that 10–15% of pregnancies will be complicated by high blood pressure [2, 3]. Up to about one-quarter of all antenatal admissions will be hypertensive related cases [2]. Over the last century, maternal mortality rates in high-income countries have steadily declined [4]. Every year about 70,000 women die and there are half a million stillbirths or neonatal deaths owing to hypertensive disorders of pregnancy (HDP)–the vast majority being in the developing world [5]. The identification of the disorder and its effective treatment play a beneficial role in pregnancy outcomes for the mother and the foetus, and hence a reduction in both maternal and perinatal mortality. Hypertensive disorders are associated with low birth weight, fetal growth restriction and prematurity which greatly contribute to perinatal morbidity and mortality [6, 7]. Many pregnancy complications that are associated with high foetal morbidity and mortality have shown gross deviations from the normal placental morphology and anatomy [8, 9]. With the placenta serving as the image for the health status of the mother and foetus, complications like hypertension in pregnancy has reflected in the placenta in a significant way, either microscopically or macroscopically [10, 11].

Pregnancies that are complicated by hypertension have been known to record higher incidence of neonatal morbidity compared to pregnancies with normal blood pressure. Pregnancies with hypertensive disorders are prone to a higher risk of preterm deliveries and low birth weights compared to healthy pregnancies [12]. The risk of HDP occurs mostly among mothers affected with severe chronic hypertension as well as those with superimposed preeclampsia on chronic hypertension [13]. The objective of this was to determine the prevalence of HDP, the morphological variations of human placenta in HDP, and maternal and neonatal outcomes in HDP.

## Materials and methods

This was a prospective case-control study, conducted at the maternity block of Komfo Anokye Teaching Hospital (KATH) in Kumasi, Ghana, during the period of February 2018 to July 2018. Samples for the study were collected following approval from the Research and Development Unit, KATH and the Committee on Human Research, Publication and Ethics (CHRPE)-KNUST. With a total of 338 pregnant women evaluated, 214 participants with complete records were available for analysis during this study period. While ensuring confidentiality, the study protocol was explained to each individual in detail and those who gave either written or verbal informed consent were included in the study. Informed consent was obtained from the guardian or next of kins of study participants who had difficulty giving informed consent because of their health status. Patients were also informed that non participation or withdrawal from the study would have no effect on the standard management of their respective medical treatment at the hospital. The Patients’ obstetric history were reviewed and those with blood pressure ≥140/90 mm Hg, with or without proteinuria diagnosed after the 20^th^ week of gestation, who developed hypertension within 48 hours after delivery, with absence of other serious diseases or congenital malformations, and with singleton pregnancy, were included in the study. Mothers with associated medical problems other than hypertension and those without antenatal records were excluded from the study.

The mothers with hypertensive disorders of pregnancy were divided into five groups according to the classification system developed by the Working Group of the national high blood pressure education program [14]. These are chronic hypertension, gestational hypertension, preeclampsia, eclampsia, and preeclampsia superimposed on chronic hypertension.

Maternal parameters recorded/measured included the age of participants, body mass index, the final blood pressure reading before and after delivery, parity, occupation and level of income, educational level, ethnicity, other previous obstetric medical history, and booking status. Blood pressure of participants were recorded as a clinical routine using OMRON (HEM-907) digital-portable automated blood pressure recorder with the woman sitting down and the feet resting on a flat surface. The third trimester blood pressure of participants were measured on three different visits before delivery. Also, the blood pressure of participants were measured after delivery. The mode of delivery (vaginal or caesarean section), maternal presentation, and the degree of tear of maternal perineum were also recorded.

The detailed assessment of placenta was accurately done using freshly delivered placentae from both the HDP group (hypertensive mothers) and the control group (normotensive mothers) at the labour ward, A1 HDU, and the theatre. Placentae were obtained soon after delivery using a clean placenta bowl or kidney dish. Each placenta sample was labelled with ID number that corresponded with the number indicated for their respective newborn and the mother.

Inspection of placenta was done to see if there was any torn tissue to suggest retained tissue. The foetal membranes were then inspected along the edges of the placenta. The foetal surface of each placenta was examined thoroughly, the state of membranes as well as the presence of any chorionic or subamniotic haematoma were noted. Examination of maternal surface was also done for the presence of retroplacental haematoma, calcification and infarction. The umbilical cord insertion types and cord vessel numbers, along with any umbilical cord abnormalities were recorded. The cord length was measured with Dritz C150 fiberglass measuring tape (Prym consumer USA Inc.).

Freshly delivered placentae were weighed at the ward using a highly sensitive mechanical kitchen scale (Zhongshan Camry Electronic Co. Model: KCH) graduated from 0–5 kg. The foetal surface of each placenta was well cleaned and then placed on a clean white rectangular board and the placental shape was observed and described as either round, oval, or irregular. The measurement of placenta diameter was done using a Dritz C150 fiberglass measuring tape (Prym consumer USA Inc.). Four different angles of each placenta were measured and the mean determined. This was done due to the fact that many of the placentae upon examination were not round in shape but rather ovoid or irregular in shape (particularly hypertensive placentae) making it impossible to take a single reading. The thickness of placenta was determined using the toothpick method [15]. This was done by piercing the placentae from the chorionic plate to the basal plate at both the centre and near the edge, with each placenta placed on the foetal surface. The values were transferred onto a clear ruler 30 cm/12 inches (Helix China Inc.) calibrated in centimetres and their averages computed to determine the placental thickness. The water displacement method was adopted in measuring the placenta volume using a calibrated one-litre beaker. With this method, the actual volume of placenta was determined as, Volume = Vol_2_ –Vol_1_, where Vol_2_ is for final volume (placenta volume + water volume) and Vol_1_ is for water volume only.

Gestational age was expressed as beginning from the last date of menstruation proven by preliminary examination with ultrasound scan. On the basis of gestational age, the infants were categorized into 3 groups: *Term infants* were those with gestational age between 37 to 42 weeks, *preterm babies* included infants with gestational age <37 weeks and *post term babies* had gestational age >42 weeks. Low birth weight (LBW) was specified for birth weight (BW) <2.5 kg, very low birth weight (VLBW) as BW <1.5 kg, and extremely low birth weight (ELBW) as BW <1 kg [16]. The standard body length was defined as length of baby ranging from 46.9 to 54.9 cm [17]. The standard head circumference was defined as circumference of the head of baby ranging from 33 to 37 cm [18]. The standard abdominal circumference was defined as the circumference of the abdomen of baby ranging from 31 to 33 cm [16].

Data was analyzed using Statistical Package for Social Sciences (SPSS) version 23.0. Statistical analysis was performed with student t-test, and chi-square test. P value equal to or less than 0.05 was considered statistically significant.

## Results

**Fig 1:**
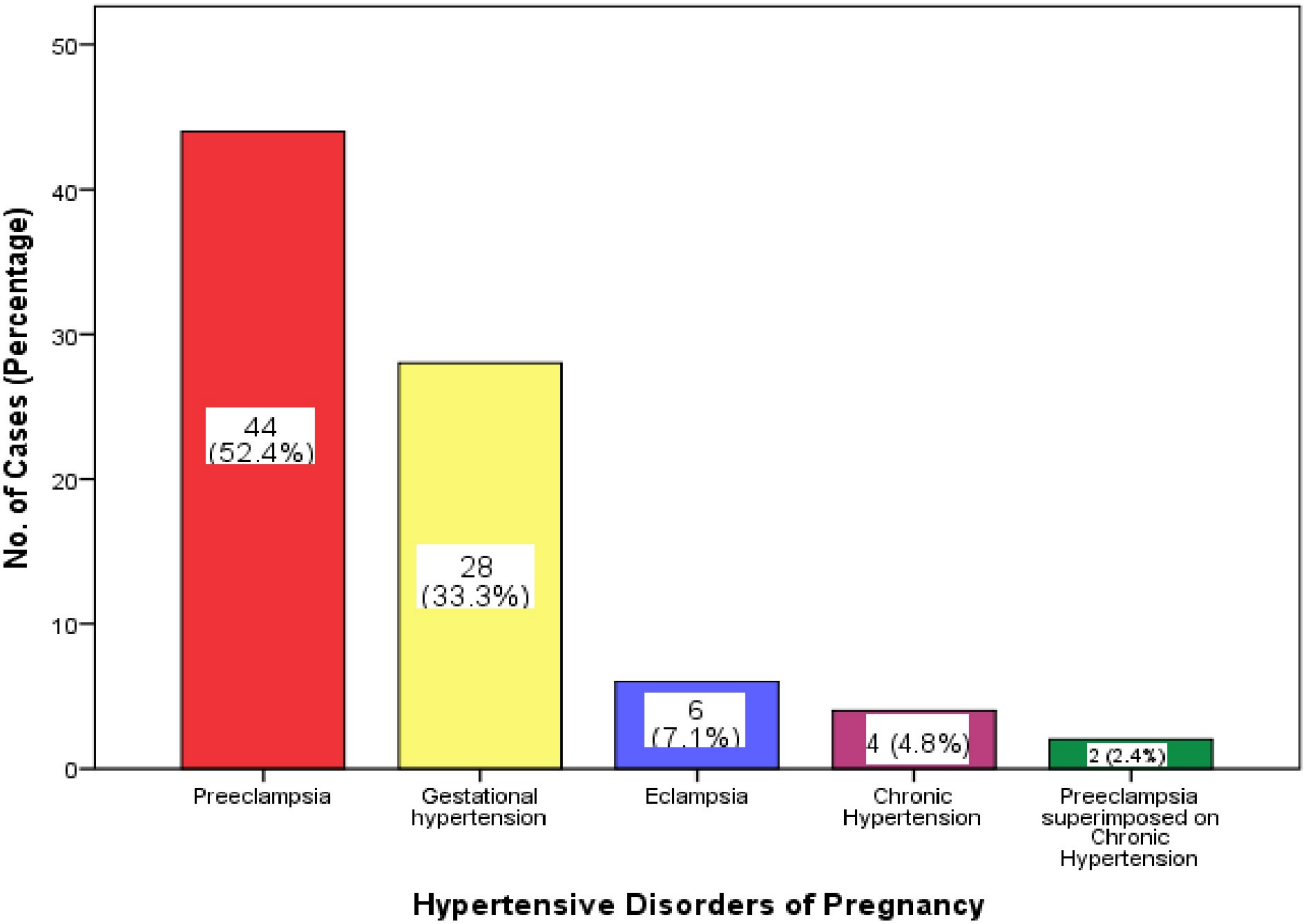
Distribution of cases HDP group according to type of hypertension.

Eighty four (39.25%) out of the 214 study population had hypertensive disorders of pregnancy (HDP). This indicates 393 out of every 1,000 patients had HDP. Of the 84 deliveries with hypertensive disorders of pregnancy, 28 (33.3%) had gestational hypertension, 44 (52.4%) had preeclampsia, 6 (7.1%) had eclampsia, 4 (4.8%) had chronic hypertension, and 2 (2.4%) had preeclampsia superimposed on chronic hypertension (Fig 1).

The mean age of the hypertensives was 29.85 years. Among the hypertensives, 31 (36.9%) were in the age group 30–34 years, and 25 (29.8%) were in the age group 25–29 years. There was a significant difference in weight (p = 0.001) and height (p = 0.025) between the hypertensives and normotensives, but the difference in BMI observed between them was not significant (p = 0.090) (Table 1). The study showed that 42 (50.0%) of the hypertensives and 57 (43.8%) normotensives were obese.

**Table 1:** Distribution according to maternal age, weight, height, and BMI.

Variable							
Age (years)	Frequency		Mean±SD		‘t’	df	p-value
	Control	HDP	Control	HDP			
≤19	13	2					
20–24	19	13					
25–29	41	25					
30–34	36	31	28.31±0.54	29.85±0.61	1.845	212	0.067
35–39	15	7					
≥40	6	6					
MW (kg)	130	84	72.79±13.10	78.84±12.46	3.368	212	0.001
MH (m)	130	84	1.58±0.08	1.60±0.07	2.255	212	0.025
BMI (kg/m^2^)	130	84	29.34±6.22	30.74±5.30	1.705	212	0.090

MW = Maternal Weight, MH = Maternal Height, BMI = Body Mass Index, SD = Standard Deviation, p = the p value.

Forty-eight (57.2%) of the hypertensives were multiparous, 19 (22.6%) primiparous and the remaining 17 (20.2%) were nulliparous, while 59 (45.4%) of the normotensives were multiparous, 32 (26.4%) were primiparous, and the remaining 39 (17%) were nulliparous. There was no significant difference in maternal parity between the hypertensives and normotensives (p = 0.324) (Table 2).

**Table 2:** Maternal parity.

Parity	Frequency		Percentage		Sum of Frequencies	χ2	df	p-value
	Control	HDP	Control	HDP				
Para-0	39	17	30.0	20.2	56			
Para-1	32	19	24.6	22.6	51			
Para-2	29	22	22.3	26.2	51	3.476	3	0.324
Para>3	30	26	23.1	31.0	56			
Total	130	84	100.0	100.0	214			

Majority (67.9%) of women with hypertensive disorders of pregnancy presented to the hospital (KATH) with headache as the chief complaint. Rare symptoms that were observed from some of the patients were abdominal pain and swollen feet. The high BP readings that were recorded in all the women with HDP persisted throughout the pregnancy period ie from the time of diagnosis to delivery. There was a slight or gradual decrease after delivery. During their hospitalization, the increases in diastolic blood pressure (DBP) corresponded with the systolic blood pressure (SBP) for both groups. The SBP was used as the major preliminary criteria for identifying the HDP group. There was a significant difference in the mean systolic BP (p = 0.001) and pulse rate (p = 0.001) of the hypertensives and normotensives before and after delivery (Table 3). The averages for the minimum and maximum values, and mean of third trimester systolic BP of participants measured on three different visits in each study group before delivery after delivery are indicated in Table 3.

**Table 3:** Blood pressure of participants.

	SBP before delivery		SBP after delivery	
	Control	HDP	Control	HDP
Minimum	96	125	90	111
Maximum	128	240	110	185
Mean±SD	120.16±12.39	161.14±19.37	110.96±12.56	148.20±16.64
‘t’	16.584		16.111	
p-value	0.001		0.001	

SBP = Systolic Blood Pressure, DBP = Diastolic Blood Pressure.

Women with chronic hypertension had a positive family history of hypertension and their high BP was present either pre-pregnancy or at <20^+0^ weeks’ gestation. Gestational hypertensive women recorded high BP at ≥20^+0^ weeks’ gestation. A large number of patients with preeclampsia (especially severe forms) presented with nausea and vomiting, epigastric pain, and visual disturbances like blindness. The eclampsia patients had the symptoms of preeclampsia associated with either mild or severe seizures. Pregnant women diagnosed of chronic hypertension with superimposed preeclampsia had chronic high BP with proteinuria, and a retrogression of blood pressure control. Women with eclampsia recorded the highest mean systolic BP (180.67 mm Hg), followed by those with preeclampsia superimposed on chronic hypertension (178.50 mm Hg), and the least was recorded in chronic hypertensive women (157.50 mm Hg). The mean systolic BP that was recorded for each of the 5 HDP subtypes were comparatively higher than that of the normotensive mothers (Table 4).

**Table 4:** Systolic BP readings of study participants.

Type of HDP	Mean Systolic BP (mm Hg)	
	Before delivery	After delivery
Chronic Hypertension	157.50	146.25
Gestational Hypertension	158.67	146.43
Preeclampsia	159.75	147.52
Eclampsia	180.67	158.67
Preeclampsia Superimposed on Chronic Hypertension	178.50	160.50
Control	120.16	110.96

The number of hypertensives in whom labour was induced, who had their babies delivered by caesarean section, and who were admitted after they had given birth, were significantly (p = 0.001) higher than that of normotensives who underwent similar procedures (Table 5). Four stillbirths were recorded in the hypertensives compared to zero in the normotensives. The number of babies delivered by the hypertensives who were admitted to the NICU was significantly (p = 0.001) higher than those delivered by normotensives. There was however, no significant differences in preterm deliveries, foetal presentation, and degree of maternal tear, between the two groups (Table 5).

**Table 5:** Delivery outcomes of study participants.

Delivery outcome	Component	Control		HDP		‘χ2’	df	p-value
		n	%	n	%			
Induction of labour	Yes	55	42.3	84	100.0			
	No	75	57.7	0	0.0	74.610	1	0.001
Term of baby	Pre-term	36	27.7	26	31.0			
	Term	94	72.3	58	69.0	0.264	1	0.608
Mode of delivery	Vaginal	128	98.5	37	44.0			
	C/S	2	1.5	47	56.0	85.581	1	0.001
Presentation	Cephalic	126	96.9	8	92.9			
	Breech	4	3.1	6	7.1	1.894	1	0.169
Perineum of mothers	Intact	74	57.4	47	56.0			
	Tear	55	42.6	37	44.0	0.020	1	0.886
Type of birth	Live	130	100.0	80	95.2			
	IUFD	0	0.0	4	4.8	6.308	1	0.012
NICU admissions	Yes	27	20.8	37	44.0			
	No	103	79.2	47	56.0	13.191	1	0.001
Mothers admitted after delivery	Yes	1	0.8	84	100.0			
	No	129	99.2	0	0.0	209.856	1	0.001

NICU = Neonatal Intensive Care Unit, C/S = Caesarean Section, IUFD = Intrauterine Foetal Death, p = the p value.

The frequency of placental haematoma, placental infarction, and placental calcification in the hypertensives were significantly (p = 0.001) higher than that of the normotensives. Thirty (35.7%) of the placentae of the hypertensives were round shaped, compared to 103 (79.2%) of the normotensives. Forty one (48.8%) of the placentae of the hypertensives were oval shaped, compared to 16 (12.3%) of the normotensives. The number of irregular shaped placentae were 13 (15.5%) and 11 (8.5%) for the hypertensives and normotensives respectively (Table 6).

**Table 6:** Gross morphology of placenta of study participants.

Parameter	Control		HDP		p-value
	Frequency	%	Frequency	%	
Haematoma					
Yes	5	3.8	28	33.3	
No	125	96.2	56	66.7	0.001
Infarction					
Yes	12	9.2	30	35.7	
No	118	90.8	54	64.3	0.001
Calcification					
Yes	43	33.1	51	60.7	
No	87	66.9	33	39.3	0.001
Placental Shape					
Round	103	79.2	30	35.7	
Oval	16	12.3	41	48.8	0.01
Irregular	11	8.5	13	15.5	

The mean placental weight (p = 0.01), placental volume (p = 0.001), placental diameter (p = 0.03), and placental thickness (p = 0.001) of the hypertensives were significantly lower than those of the normotensives (Table 7).

**Table 7:** Placental indices of the participants.

Variable	Minimum		Maximum		Mean±SD		‘t’	‘df’	p-value
	Control	HDP	Control	HDP	Control	HDP			
PW (kg)	0.20	0.20	0.74	0.60	0.56±0.13	0.49±0.18	3.518	212	0.01
PV (L)	0.22	0.18	0.77	0.65	0.54±0.11	0.46±0.15	4.577	212	0.001
PD (cm)	15.00	14.00	26.00	23.00	20.01±1.95	19.23±1.96	3.012	212	0.03
PT (cm)	1.10	0.90	2.70	2.30	2.09±0.29	1.88±0.33	4.834	212	0.001

PPW = Placental Weight, PV = Placental Volume, PD = Placental Diameter, PT = Placental Thickness SD = Standard Deviation, p = the p value.

Thirty one (36.9%) placentae of the hypertensives had central cord insertion, 28 (33.3%) were eccentric, 23 (27.4%) were marginal, and 2 (2.4%) were velamentous. Eighty five (65.4%) of the placentae of the normotensives had eccentric cord insertion, 21 (16.2%) were central, 22 (16.9%) were marginal and 2 (1.5%) were velamentous. Although majority of the umbilical cords from both groups had 3 vessels, the mean number of umbilical cords that had 3 vessels were significantly (p = 0.001) lower in the hypertensives, compared to the normotensives (Table 8).

**Table 8:** Umbilical cord indices.

Parameter	Control		HDP		p-value
	Frequency	%	Frequency	%	
**Umbilical Cord Insertion**					
Velamentous	2	1.5	2	2.4	
Central	21	16.2	31	36.9	0.001
Eccentric	85	65.4	28	33.3	
Marginal	22	16.9	23	27.4	
**Umbilical Cord Vessel Number**					
Three	121	93.1	59	70.2	
Two	8	6.2	21	25.0	0.001
One	1	0.8	4	4.8	
**Umbilical Cord Length (cm)**					
Short	31	23.9	45	53.6	
Normal	80	61.5	37	44.0	0.001
Long	19	14.6	2	2.4	

Majority (53.6%) of the umbilical cords of the hypertensives were short while majority (61.5%) of the umbilical cords of the normotensives had normal lengths. The mean cord length of the hypertensives (39.11±13.05 cm) was significantly lower than that of the normotensives (51.01±16.13 cm) (p = 0.001) (Table 8).

The mean birth weights, birth lengths, head circumferences, and abdominal circumferences of the neonates of the hypertensives were significantly lower than that of the neonates of the normotensives (p = 0.001) (Table 9). Additionally, the Apgar score at the 5^th^ minute of birth of neonates of the hypertensives was significantly lower than that of the neonates of the normotensives (p = 0.001) (Table 9).

**Table 9:** Neonatal indices of study participants.

Parameter	Minimum		Maximum		Mean±SD		‘t’	‘df’	p-value
	Control	HDP	Control	HDP	Control	HDP			
BW (kg)	0.90	0.72	4.41	3.80	3.07±0.55	2.70±0.75	4.197	212	0.001
BL (cm)	38.0	33.0	57.0	53.0	48.27±4.46	45.18±4.42	4.976	212	0.001
HC (cm)	28.0	24.0	41.0	40.0	34.68±2.45	33.14±3.81	3.594	212	0.001
AC (cm	37.0	20.0	40.0	21.0	32.60±3.11	30.76±3.90	3.814	212	0.001
AS	^3^/_10_	^0^/_10_	^10^/_10_	^9^/_10_	8.40±1.19	7.70±1.75	3.472	212	0.001

BW = Birth Weight, BL = Baby Length, HC = Head Circumference, AC = Abdominal Circumference, AS = Apgar Score, SD = Standard Deviation, p = the p value.

## Discussion

HDP has become a major health issue worldwide, and the prevalence varies from one country to another as well as in different institutions. This study showed a HDP prevalence of 39.25% at Komfo Anokye Teaching Hospital (KATH) during the study period. There has been a reported incidence of 1.5% to 22% of all pregnancies, which is dependent upon the population sampled and the definitions used [19–23]. The variation may be due to differences in genetic factors, socioeconomic status, racial differences, and some other demographic features such as maternal age and parity [21–23]. Another reason might be the differences in terminologies used in the study methodologies. Pregnancy Induced Hypertension (PIH) for instance, has led to a significant debate with misleading account in HDP prevalence, rendering the term PIH obsolete and no longer recommended in literature [19]. Prevalence rates of 21.4%, 17%, and 16.8% have been recorded at Korle-bu Teaching Hospital in Ghana, in a tertiary referral heath facility in Nigeria, and in North West Ethiopia respectively [24–26].

The prevalence of HDP with respect to age-group distribution was at its peak in women of 40 years and over (many of them being chronic hypertensives), and the lowest number was recorded for mothers who were 19 years or below. Other studies found the highest and lowest proportion of women with HDP between 25–29 years and ≥40 years respectively [7], results inconsistent with that of the present study. Other researchers have reported an increased risk of HDP like preeclampsia in younger women who were 21 years or below [27, 28].

Most of the women with hypertensive disorders of pregnancy in this study were multiparous (parity of 3 or more), followed by primiparous women. This is similar to some studies that found a higher prevalence of HDP among women with grand multiparity (5 or more) [7, 29]. From this study, the mean of third trimester Systolic BP of participants measured on three different visits before and after delivery was significantly (p = 0.001) higher in hypertensive mothers than normotensive mothers, a result similar to findings of other studies [30, 31]. Some investigators have found that women with HDP had a mid-trimester decrease, which was followed by a progressive rise in both systolic BP and diastolic BP between 30–45 days postpartum [32, 33]. The factors influencing the development of high blood pressure may differ depending on the particular type of hypertensive disorder, the study population (ethnicity or race), family history of the individual [34], life style and eating habit of the individual [35], and most importantly the age and parity of the pregnant woman [36].

From the antenatal history, mothers who had higher BMI at the beginning of pregnancy or were overweight or obese during gestation, showed higher SBP and DBP values in all gestational trimesters until delivery. Contrary to the findings of this study, some previous studies have found underweight pregnant women to be at risk of hypertension development which result in delivery of preterm infants [37, 38]. Higher maternal weight before pregnancy increases the risk of late fetal death, although it protects against the delivery of a small-for-gestational-age infant [39].

In this study, most of the placentae (48.8%) of the hypertensives were oval in shape, while most of the placentae (79.2%) of the normotensives were round in shape, a finding similar to that of other studies [11, 40, 41]. One study found no significant difference (p > 0.05) between the number of different placental shapes of the hypertensives and normotensives [42], and another study found that the shape of placentae from both hypertensives and normotensives were either oval or round [43]. The significantly high incidence of placental haematoma in hypertensives compared with the normotensives in this study is consistent with the findings of other studies [6, 44]. A study has found an association between placental haematoma and low Apgar score and also an association between larger haematomas and IUFD, due in part to separation of a considerable part of the villi from the utero placental circulation [45].

The number of placentae with infarction was significantly (p = 0.001) higher in the hypertensives than the normotensives in this study. This is consistent with the results obtained by other studies [40, 46, 47]. Placental infarcts are known to have an adverse effect on growth and development of the newborns [47].

The present study also observed a significantly high placental calcification in the hypertensives compared to the normotensives. This is similar to the findings of a study which concluded that the foetal outcome in terms of birth weight of newborns to mothers having PIH and calcification of placentae was poor when compared to the control group [48]. Another study found no significant difference in the incidence of calcification in the hypertensives and normotensives [49]. It is noteworthy that calcification that is seen in the placenta shows an evidence of placental senescence or degeneration [50].

The mean placental weight, volume, thickness, and diameter for hypertensives were significantly lower than that of the control group in the present study (p < 0.05). Similar outcomes in placental parameters have been reported by other studies [11, 41, 51–54]. This study results suggest that placental weight may be a good predictor of newborn weight, because a significant linear correlation was observed for both the hypertensives (r = 0.579, p = 0.001) and the normotensives (r = 0.630, p = 0.001). Similar relations have been shown by other researchers [8, 55, 56]. The present study recorded a significant (p < 0.001) reduction in the mean central thickness of placentae in the hypertensives compared to the normotensives. This finding is consistent with that of other studies [57–62]. The results for placental volume obtained in this study was similar to that obtained by other studies [63–65].

Majority of the umbilical cord lengths in the hypertensive mothers were significantly short compared to that of the normotensives in this study. Short umbilical cord lengths are associated with a high rate of foetal abnormalities, such as abdominal wall defects and defects in the extremities and spine [66]. They are also associated with unsatisfactory foetal state, central nervous system complications, and low Apgar and IQ scores [67]. A normal umbilical cord has two arteries and a vein and is covered by Wharton’s jelly. Changes may sometimes occur during pregnancy that result in abnormal number of umbilical cord vessels [68]. The number of umbilical cords with three vessels in the hypertensives was significantly lower compared to the that of the normotensives in this study. Almost 30% of the umbilical cords of the hypertensives had less than 3 vessels compared to only 7% of the normotensives. The result of the present study is contrary to that of Saha *et al*. (2014) [69] who found 3 vessels in all their samples.

The umbilical cord insertion site to the placenta can be central, eccentric, marginal (battledore), or velamentous (membranous). More than 90% of term placentae insertions are central or eccentric. Marginal cord insertion (MCI) and velamentous cord insertions (VCI) are classified as abnormal placental cord insertions (PCI). VCI occurs in approximately 1% of singleton pregnancies and MCI in approximately 7% [66]. Eighty one percent of the umbilical cord insertions in the normotensives in this study were either central or eccentric compared to 70.2% in the hypertensives. The frequency of marginal and velamentous cord insertions was higher in the hypertensives. This finding is similar but not to the same degree as that of other studies [70, 71]. Abnormalities of the umbilical cord, related to morphology, placental insertion, number of vessels and primary tumors, can influence the perinatal outcome and may be associated with other fetal anomalies and aneuploidies [68].

The number of preterm deliveries for the hypertensive and normotensive mothers in the present study was not significantly different. A result contrary to that of Yadav *et al*. (1997) [72] who recorded significantly high numbers of preterm deliveries among the hypertensives compared to the normotensives. The need to induce labour or perform a caesarean section on the mothers was significantly higher (p = 0.001) in the hypertensives than the normotensives. The still birth rate was also significantly higher (p = 0.012) in the hypertensives. These findings are similar to that of Yadav *et al*. (1997) [72]. The number of babies born to hypertensives who needed NICU care was significantly higher compared to those of the normotensives, results similar that of other studies [72–74]. It must be stated however that the frequencies in the present study were at times higher or lower than that of these studies.

The present study showed that the foetal development rate of hypertensives could be affected by adverse maternal and placental factors. The mean birth weight, baby length, abdominal circumference, and head circumference of neonates of the hypertensives were significantly (p = 0.001) lower compared to the normotensives in this study. Similar findings of LBW babies were observed in other studies [11, 40, 41, 75]. The APGAR scores after five minutes of delivery was significantly (p≥0.001) lower in infants of the hypertensives compared to the infants of the normotensives. This is consistent with the findings of other studies [74, 76].

## Conclusion

The study found a high prevalence of hypertensive disorders of pregnancy at the study site. Those suffering from this condition were at a high risk of having placental abnormalities that would impact negatively on maternal and perinatal outcomes. Early detection and management of HDP should be instituted to forestall such outcomes.

## Supporting information

S1 Data (XLSX)
